# Icariin promotes directed chondrogenic differentiation of bone marrow mesenchymal stem cells but not hypertrophy *in vitro*

**DOI:** 10.3892/etm.2014.1950

**Published:** 2014-09-08

**Authors:** ZHI CONG WANG, HUI JUN SUN, KAI HUA LI, CHAO FU, MO ZHEN LIU

**Affiliations:** 1 Department of Orthopedic Surgery, First Affiliated Hospital of Dalian Medical University, Dalian, Liaoning 116011, P.R. China; 2 Department of Clinical Pharmacology, Dalian Medical University, Dalian, Liaoning 116044, P.R. China

**Keywords:** bone marrow mesenchymal stem cells, chondrogenesis, hypertrophy, icariin

## Abstract

Icariin (ICA), a Traditional Chinese Medicine, has been demonstrated to be a promoting compound for extracellular matrix synthesis and gene expression of chondrocytes. However, whether ICA can act as a substitute for or cooperate with growth factors to directly promote stable chondrogenesis of bone marrow mesenchymal stem cells (BMSCs) remains unknown. In the present study, rat BMSCs were cultivated in monolayer cultures with a chondrogenic medium containing transforming growth factor-β3 for 14 days; ICA was added to the same chondrogenic medium throughout the culture period at a concentration of 1×10^−6^ M. Cell morphology was observed using an inverted microscope, and chondrogenic differentiation markers, including collagen II, aggrecan and SRY (sex determining region Y)-box 9 (SOX9), were detected by immunofluorescence, reverse transcription-quantitative polymerase chain reaction and western blot analysis. Hypertrophic differentiation was also analyzed using collagen I gene expression and alkaline phosphatase (ALP) activity. The results revealed that ICA was effective at forming an increased number of and larger aggregates, and significantly upregulated the mRNA expression levels and protein synthesis of collagen II, aggrecan and SOX9. Furthermore, the chondrogenic medium alone caused hypertrophic differentiation through the upregulation of collagen I gene expression and ALP activity, which was not potentiated by the presence of ICA. Thus, ICA promoted directed chondrogenic differentiation of BMSCs, but had no effect on hypertrophic differentiation. The present results also suggested that ICA may be an effective accelerant of growth factors for cartilage tissue engineering by promoting their chondrogenic differentiating effects but reducing the effect of hypertrophic differentiation.

## Introduction

Cartilage defects are difficult to heal spontaneously, as cartilage tissue lacks blood vessels, nerves and lymph supplies, and the cartilage lesions do not usually reach the progenitor cells of the bone marrow (1). Severe cartilage damage caused by degeneration or excessive use may lead to osteoarthritis (OA), which causes pain, compromises mobility and poses a significant disease burden across the world (2). At present, cartilage tissue engineering is considered to be one of the most promising therapeutic approaches for the treatment of cartilage defect. The mechanism of this therapy is based on the fact that bone marrow mesenchymal stem cells (BMSCs) have the potential for multilineage differentiation, including osteogenesis, chondrogenesis and adipogenesis, as well as extensive proliferation.

Growth factors play a crucial role in the regulation of BMSC differentiation. A number of studies have demonstrated that transforming growth factor-β (TGF-β), bone morphogenetic protein (BMP) and insulin-like growth factor are able to induce chondrogenic differentiation *in vitro*, and promote the formation of cartilage-like tissue *in vivo* (3–6). However, growth factors not only upregulate the expression of hyaline cartilage-specific markers, such as collagen II, but also inevitably lead to further hypertrophic differentiation and contribute to the development of fibrous cartilage (7–10). Furthermore, the high cost, rapid degradation and easily-lost activity of growth factors limit their widespread use, particularly in clinical practice (11–13). In order to promote chondrogenesis and maintain the stable chondrogenic phenotype without hypertrophy, there is an urgent requirement to develop safe and low-cost drugs that can act as a substitute for or cooperate with growth factors (11,12).

Herba Epimedii (HEP) is a widely used traditional Chinese herb to treat osteoporosis in China, Japan and Korea (13,14). Icariin (ICA; C_33_H_40_O_15_; molecular weight, 676.65), the main pharmacologically active compound of HEP, has been suggested to be a potential accelerator for cartilage tissue engineering and a substitute for growth factors. However, these results were based on the use of chondrocytes (11,12,15). Although the application of chondrocytes in cartilage tissue engineering is relatively prevalent, several major challenges exist, including chondrocyte dedifferentiation, donor site morbidity and limited sources for harvesting cartilage tissue (1). Therefore, the present study investigated whether ICA had the potential to promote stable chondrogenesis of BMSCs without hypertrophic differentiation on the basis that the same chondrogenic medium containing TGF-β3 was added.

## Materials and methods

### Cell culture

Rat BMSCs were purchased from Cyagen Biosciences (Guangzhou, China) and characterized by specific cell surface markers, including cluster of differentiation (CD)29, CD34, CD44, CD45, CD11b and CD90. The cells were highly positive for CD29 (83.99%), CD44 (99.69%) and CD90 (95.05%), and negative for CD34 (0.62%), CD45 (0.28%) and CD11b (4.25%), and were able to differentiate into osteoblasts, chondrocytes and adipocytes. The cells were cultured in low-glucose Dulbecco’s modified Eagle’s medium (LG-DMEM; HyClone Laboratories, Inc., Logan, UT, USA) containing 10% fetal bovine serum (Hyclone Laboratories, Inc.), 10 U/ml penicillin G and 10 mg/ml streptomycin (Hyclone Laboratories, Inc.) in a 5% CO_2_ incubator at 37°C.

### Cell differentiation

To establish BMSC chondrogenesis in monolayer culture, a procedure was carried out as previously described (16). In brief, cells at passage six were seeded onto 24-well plates at a density of 1×10^4^ cells/well and cultured in LG-DMEM without chondrogenic supplements. The medium was replaced with chondrogenic medium after one day, which was then changed every two days. The chondrogenic medium contained 0.1 μM dexamethasone, 50 μg/ml ascorbate, 1% insulin-transferrin-selenium, 100 μg/ml sodium pyruvate, 40 μg/ml proline and 10 ng/ml TGF-β3 (Cyagen Biosciences). The cells were divided into three groups: i) Control (cultured with serum-free LG-DMEM only); ii) TGF-β3 (cultured with chondrogenic medium containing 10 ng/mlTGF-β3); and iii) TGF-β3 + ICA (cultured with chondrogenic medium containing 10 ng/ml TGF-β3 and 1×10^−6^ M ICA). ICA was purchased from the National Institute for the Control of Pharmaceutical and Biological Products of China (Beijing, China). The morphology of the seeded BMSCs was observed using an inverted microscope (CKX41; Olympus, Tokyo, Japan).

### Immunofluorescence

At day 14, cultured cells were washed three times with phosphate-buffered saline (PBS) and fixed for 10 min with 4% paraformaldehyde. Specimens were blocked with 5% bovine serum albumin for 1 h and incubated at 4°C overnight with the following primary antibodies: Anti-collagen II (1:100; GeneTex, Irvine, CA, USA), anti-aggrecan (1:200; Millipore, Billerica, MA, USA) and anti-SRY (sex determining region Y)-box 9 (SOX9) (1:200; Abcam, Cambridge, UK). Subsequent to washing three times with PBS, the cells were incubated with fluorescent secondary antibodies (Beyotime Institute of Biotechnology, Shanghai, China) for 2 h. For nuclear staining, DAPI (Beyotime Institute of Biotechnology) was applied for 3 min and subsequently observed under a fluorescence microscope (Leica DM 4000 B; Leica Microsystems, Wetzlar, Germany). Cell counting of 1,000 cells in five randomly selected fields was performed by Image-Pro Plus 6.0 software (Media Cybernetics, Inc. Rockville, MD, USA) and the percentage of positively stained cells was calculated as the ratio of the number of positive cells to the total number of DAPI-positive cells.

### Reverse transcription-quantitative polymerase chain reaction (RT-qPCR)

Total RNA was isolated using RNAiso Plus (Takara Bio, Inc., Dalian, China), following the manufacturer’s instructions, and was quantified by absorbance analysis at 260 nm. cDNA was synthesized using a Primescript^™^ RT reagent kit with gDNA Eraser (Takara Bio, Inc.). The RT-qPCR reactions were performed with an ABI PRISM^®^ 7500 Real-Time PCR System (Applied Biosystems^®^, Invitrogen Life Technologies, Carlsbad, CA, USA) with SYBR Premix Ex Taq^™^ (Takara Bio, Inc.). Subsequent to adding equal amounts of cDNA and specific primers to the mix, initial denaturation was carried out at 94°C for 30 sec, followed by 40 cycles of denaturation at 94°C for 5 sec, annealing at 60°C for 15 sec and extension at 72°C for 10 sec. The primers used in RT-qPCR analysis were as follows: Collagen, type II, α1 (Col2a1) forward, 5′-CGCCACGGTCCTACAATGTC-3′ and reverse, 5′-GTCACCTCTGGGTCCTTGTTCAC-3′; collagen, type I, α1 (Col1a1) forward, 5′-GCCTCCCAGAACATC ACCTA-3′ and reverse, 5′-GCAGGGACTTCTTGAGGTTG-3′; aggrecan forward, 5′-TGGCATTGAGGACAGCGAAG-3′ and reverse, 5′-TCCAGTGTGTAGCGTGTGGAAATAG-3′; SOX9 forward, 5′-GCAGAGACTGAAGACCCTACACAGA-3′ and reverse, 5′-GAGGCAACTTCACGCTGCAA-3′; GAPDH forward, 5′-TATGACTCTACCCACGGCAA-3′ and reverse, 5′-ATACTCAGCACCAGCATCACC-3′. The levels of mRNA expression were analyzed by the 2^−ΔΔCt^ method using GAPDH as a control.

### Western blot analysis

Cell extracts were prepared using a Protein Extraction kit (Nanjing KeyGen Biotech. Co., Ltd., Nanjing, China), and protein concentrations were measured with a bicinchonic acid protein assay kit (Boster Biological Technology Co., Wuhan, China). The protein samples were denatured at 100°C for 5 min and separated by SDS-PAGE. The proteins were subsequently transferred to polyvinylidene difluoride membranes and incubated at 4°C overnight with the primary antibodies: Anti-collagen II (1:1,000; GeneTex), anti-aggrecan (1:1,000; Millipore), anti-SOX9 (1:500; Abcam) and anti-GAPDH (1:1,000; Santa Cruz Biotechnology, Inc., Santa Cruz, CA, USA). Subsequent to being washed three times with PBS, the membranes were incubated with anti-mouse or anti-rabbit secondary antibodies (1:1,000; ZSGB-BIO, Beijing, China) for 2 h and visualized with a BeyoECL Plus kit (Beyotime Institute of Biotechnology).

### Alkaline phosphatase (ALP) activity

ALP activity was measured in the culture supernatants as previously described (7). Briefly, the media were replaced with the phenol red free equivalent at day 12. The culture supernatants were collected after two days and centrifuged at 1,400 × g for 10 min. Soluble ALP activity was detected with an alkaline phosphatase kit (Nanjing Jiancheng Bioengineering Institute, Nanjing, China).

### Statistical analysis

Data are presented as the mean ± standard deviation and were analyzed using SPSS 16.0 software (SPSS, Inc., Chicago, IL, USA). The differences between the multiple group comparisons were evaluated by one-way analysis of variance followed by Tukey’s test. P<0.05 was considered to indicate a statistically significant difference, and P<0.01 was considered to indicate a marked statistically significant difference.

## Results

### ICA affects BMSC morphology during chondrogenesis

**Figure 1:**
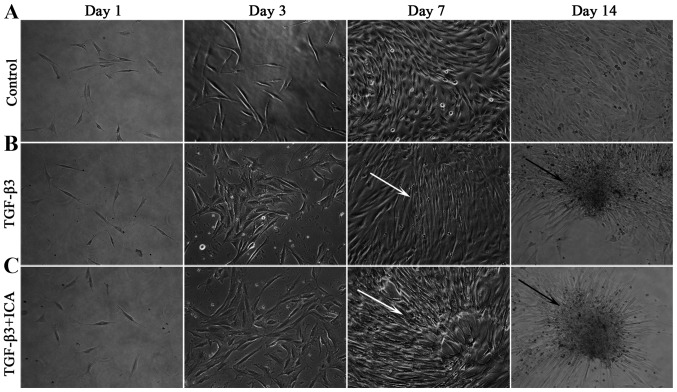
Effects of ICA on cell morphology during chondrogenic differentiation at days 1, 3, 7 and 14. (A) Control group; (B) TGF-β3 group; (C) TGF-β3 + ICA group (magnification, ×100). White arrows, mono-layered aggregates; black arrows, multi-layered aggregates; ICA, icariin; TGF-β3, transforming growth factor-β3.

Throughout the culture period, the BMSCs cultured in LG-DMEM grew as monolayers and exhibited a characteristic spindle-like, fibroblastic morphology (Fig. 1A). By contrast, cells induced with the chondrogenic medium began to lose the typical morphology at day 3 and compacted to form a few mono-layered aggregates and small multi-layered aggregates at days 7 and 14, respectively (Fig. 1B). Notably, a number of mono-layered aggregates and large multi-layered aggregates were visible at days 7 and 14 in the presence of 1×10^−6^ M ICA (Fig. 1C).

### ICA promotes the chondrogenic differentiation of BMSCs

**Figure 2:**
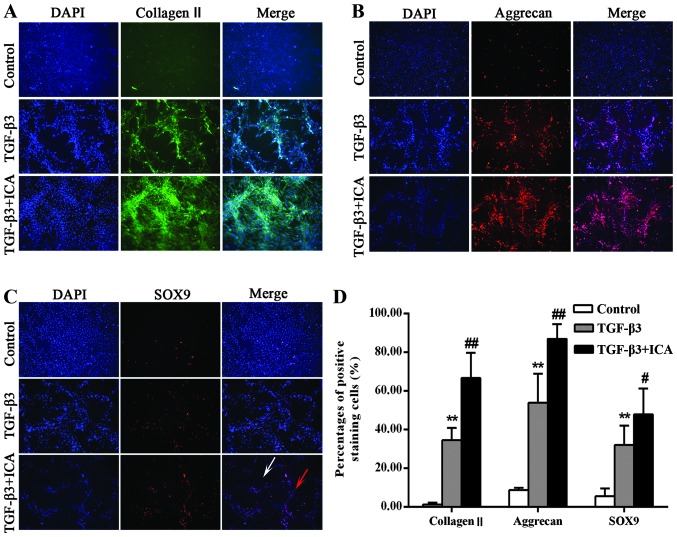
Effects of ICA on cartilage-specific markers detected by immunofluorescence in cultured cells at day 14. (A) Cells were stained for collagen II (green) and nuclei were stained with DAPI (blue). (B) Cells were stained for aggrecan (red). (C) Cells were stained for SOX9 (red). (D) The percentage of collagen II-, aggrecan- and SOX9-positive cells was calculated. ^**^P<0.01 vs. the control group; ^##^P<0.01 and ^#^P<0.05 vs. the TGF-β3 group. Magnification, ×100. Red arrow, stain for SOX9 in the aggregates; white arrow, stain for SOX9 in the single cells; ICA, icariin; TGF-β3, transforming growth factor-β3; SOX9, SRY (sex determining region Y)-box 9.

The effects of ICA on cartilage-specific markers are shown in Fig. 2. Collagen II and aggrecan are the major structural components of articular cartilage extracellular matrix (ECM). Therefore, collagen II and aggrecan were observed in the chondrogenic medium with and without ICA. They were mainly present at the center of the aggregates but were barely detectable in the control group. When treated with ICA, the cells synthesized more collagen II and aggrecan compared with the TGF-β3 group (Fig. 2A and B). A semi-quantitative assessment also verified this finding (Fig. 2D). In the control group, only 1.31±0.89 and 8.77±1.21% of the cells stained positively for collagen II and aggrecan, respectively, while the percentage of positively stained cells in the TGF-β3 group was significantly increased to 34.62±6.34 and 47.37±8.39%, respectively. Furthermore, treatment with ICA markedly increased the positive staining percentages to 66.70±13.12 and 86.96±7.68% for collagen II and aggrecan, respectively.

SOX9 is considered to be an early chondrogenic marker that induces the synthesis of collagen II and aggrecan. The present study further investigated whether ICA was able to promote the expression of SOX9 and subsequently induce chondrogenesis. As shown in Fig. 2C and D, the TGF-β3 + ICA group exhibited more intense staining of SOX9 and an increased percentage of positively stained cells (47.87±13.32%) compared with the TGF-β3 group (32.02±9.42%). Notably, the majority of cells stained positively within the aggregates, while less staining was detected in the single cells, suggesting that it is necessary for BMSCs undergoing chondrogenic induction to form aggregates and that ICA can induce the chondrogenesis.

**Figure 3:**
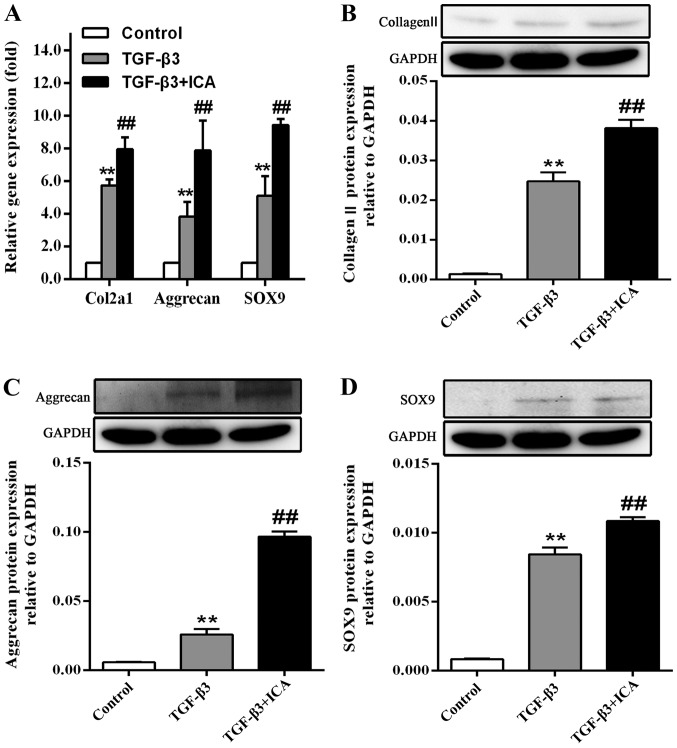
Effects of ICA on gene expression levels and protein synthesis of cartilage-specific markers. (A) Gene expression levels were analyzed by the reverse transcription-quantitative polymerase chain reaction. (B–D) Protein synthesis of (B) collagen II, (C) aggrecan and (D) SOX9 was analyzed by western blot analysis. ^**^P<0.01 vs. the control group; ^##^P<0.01 vs. the TGF-β3 group. ICA, icariin; TGF-β3, transforming growth factor-β3.

As shown in Fig. 3A, the effect of ICA on the gene expression levels of Col2a1 (the collagen II-encoding gene), aggrecan and SOX9 following BMSC culture for 14 days was also investigated. RT-qPCR analysis demonstrated that treatment with ICA produced a significant 1.38-fold increase in Col2a1 expression, a 2.06-fold increase in aggrecan expression and a 1.85-fold increase in SOX9 expression, in comparison with the TGF-β3 group.

It was also observed that BMSCs cultured in the absence of chondrogenic medium did not synthesize collagen II, aggrecan and SOX9; however, the protein expression levels were markedly increased in the TGF-β3 group. Furthermore, ICA treatment significantly promoted the synthesis of these cartilage-specific markers during the chondrogenic induction, which was consistent with the results of immunofluorescence analysis and RT-qPCR (Fig. 3B–D).

### ICA does not promote hypertrophic differentiation of BMSCs

**Figure 4:**
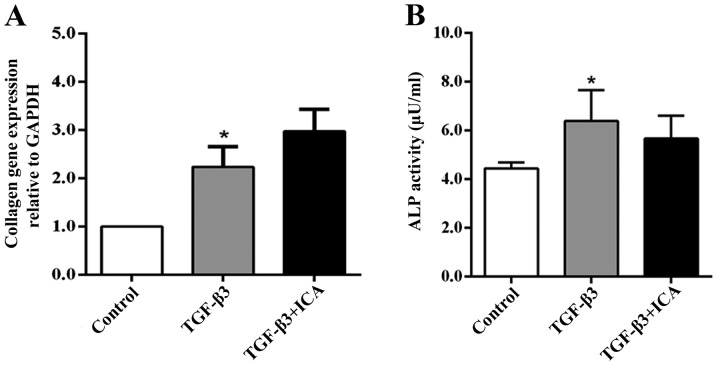
Effects of ICA on hypertrophic differentiation markers. (A) Collagen, type I, α1 gene expression was analyzed by the reverse transcription-quantitative polymerase chain reaction. (B) Soluble ALP activity was quantified in culture supernatants at day 14.^*^P<0.05 vs. the control group. ICA, icariin; TGF-β3, transforming growth factor-β3; ALP, alkaline phosphatase.

In order to demonstrate the effect of ICA on the hypertrophic differentiation of BMSCs, Col1a1 gene expression and ALP activity, markers of hypertrophy or dedifferentiation of chondrocytes, were examined following 14 days of culture. RT-qPCR analysis revealed that the expression levels of Col1a1 in the TGF-β3 group were increased compared with those in the control group. However, treatment with ICA did not significantly upregulate the expression of Col1a1 compared with the TGF-β3 group (Fig. 4A). Similarly, only the TGF-β3 group exhibited significantly higher ALP activity than the control group, while a reduction in ALP activity was detected in the ICA treatment group compared with the TGF-β3 group, although the difference was not significant (Fig. 4B). These results indicated that ICA did not potentiate BMSC hypertrophic differentiation concomitantly with promoting chondrogenesis.

## Discussion

ICA has been effectively used in the treatment of osteoporosis, brain injury and cardiovascular disease (14,17,18). In particular, the long-term development of the drug and extensive case record of its safe usage have made it an attractive treatment option (12). When chondrocytes were cultured as the source cells, ICA was revealed to promote cartilage ECM synthesis and the expression levels of chondrogenic genes. ICA also improved the restoration efficiency of supercritical-sized osteochondral defects and enhanced the integration of newly formed cartilage with subchondral bone in a rabbit model (11,12). Several studies have shown that ICA is a safe and strong chondrocyte anabolic agent, which can enhance chondrocyte proliferation, attenuate lipopolysaccharide-induced inflammatory responses and reduce ECM degradation through the inhibition of nitric oxide, matrix metalloproteinase synthesis and cathepsin K activity (15,19). Furthermore, Zhang *et al* (20) reported that ICA reduced the activity of the transcription factor nuclear factor-κB in an inflammatory model induced by tumor necrosis factor-α and also protected chondrocytes from damage due to OA. There are certain potential molecular mechanisms that explain these effects. For instance, ICA not only enhances the expression and secretion of various growth factors, including BMP-2 and TGF-β1, but also upregulates the expression levels of Drosophila mothers against decapentaplegic (Smad) proteins, including Smad1, Smad4 and Smad5, which are key regulators specific to TGF-β1 activation affecting chondrogenic genes (21,22). There has been considerable evidence to suggest that BMP and TGF-β signals play a pivotal role in chondrogenic differentiation (3,5). Hypoxia-enhanced chondrogenesis of BMSCs occurs via the activation of the mitogen-activated protein kinase (MAPK) P38 pathway (23). Furthermore, ICA stimulates P38-MAPK activation in cardiomyocyte differentiation or neuronal protection (17,18). Thus, we hypothesized that ICA may promote the directed chondrogenic differentiation of BMSCs.

Glennon-Alty *et al* (16) reported that MSCs initially grew as a monolayer and subsequently compacted to form mono-layered and multi-layered aggregates; collagen II tended to be expressed at the center of the aggregates. A number of other studies also demonstrated that BMSCs induced with chondrogenic medium developed into a round phenotype and aggregated spontaneously into spheroid- or rod-like cell agglomerates. The formation of the aggregates exhibited a more intense staining for chondrogenic matrix deposition (24,25). Consistent with these studies, the present data revealed that treatment with ICA led to the formation of an increased number of and larger aggregates. More intense staining for collagen II, aggrecan and SOX9 was also visible within the aggregates. Notably, less staining for SOX9 was detectable in the single cells outside the aggregates, which may be due to the fact that chondrogenic differentiation requires the cell-cell and cell-matrix contacts created within the aggregates (25).

Previous studies have revealed that ICA significantly affects the expression levels of cartilage-specific genes (Col2a1, aggrecan and SOX9) and leads to higher levels of aggrecan production (11,12). Similarly, it was observed in the present study that ICA notably upregulated the expression levels of these genes, increased the staining intensity and protein levels of collagen II, aggrecan and SOX9. Hattori *et al* (26) demonstrated that SOX9 was highly expressed in chondrocytes of the prehypertrophic zone and was able to directly suppress hypertrophic differentiation. In the present study, upregulation of the expression of the SOX9 gene and protein indicated that ICA either delayed or prevented the BMSCs from dedifferentiating into a hypertrophic phenotype, although the same chondrogenic medium containing TGF-β3 was added. Aggrecan in the ECM maintained the structural integrity of the articular cartilage and allowed the normal biological function of the chondrocytes, including adhesion, migration, proliferation and differentiation, to be retained. In particular, aggrecan expression and synthesis was greatly enhanced in the ICA treatment group, consistent with another study (11).

ALP is a marker of hypertrophic differentiation and collagen I is a the marker of dedifferentiated chondrocytes. A number of studies have demonstrated that the use of growth factors, including TGF-β3, produces cartilage-specific matrix but also causes hypertrophy through the upregulation of hypertrophic markers (7–10). In the present study, it was observed that chondrogenic medium alone led to higher collagen I expression and increased ALP activity. However, the presence of ICA did not potentiate the effect of the growth factors on hypertrophic differentiation while producing stronger chondrogenic differentiating effects. Similarly, other studies found that ICA downregulated Col1a1 gene expression. Collagen type X expression, another marker of hypertrophic differentiation, was barely detectable in the culture medium (11,12).

In conclusion, the present study investigated the effects of ICA on BMSC phenotypes, including cell morphology and ECM synthesis, and the expression levels of cartilage-specific genes *in vitro*. It was demonstrated that ICA not only promoted the formation of larger aggregates but also enhanced ECM synthesis and increased the expression levels of cartilage-specific genes. However, ICA exhibited no effect on hypertrophic differentiation, suggesting that ICA may be a potential promoting compound for cartilage tissue engineering and may reduce the effect of growth factors that contribute to further hypertrophic differentiation.
